# Experimental Handling Challenges Result in Minor Changes in the Phagocytic Capacity and Transcriptome of Head-Kidney Cells of the Salmonid Fish *Coregonus maraena*

**DOI:** 10.3389/fvets.2022.889635

**Published:** 2022-05-03

**Authors:** Joan Martorell-Ribera, Dirk Koczan, Marzia Tindara Venuto, Torsten Viergutz, Ronald M. Brunner, Tom Goldammer, Ulrike Gimsa, Alexander Rebl

**Affiliations:** ^1^Fish Genetics Unit, Institute of Genome Biology, Research Institute for Farm Animal Biology (FBN), Dummerstorf, Germany; ^2^Psychophysiology Unit, Institute of Behavioural Physiology, FBN, Dummerstorf, Germany; ^3^Immunology Unit, Department of Pathology and Experimental Therapy, School of Medicine and Health Sciences, Universitat de Barcelona – UB, L'Hospitalet de Llobregat, Barcelona, Spain; ^4^Core Facility for Microarray Analysis, Institute of Immunology, University of Rostock, Rostock, Germany; ^5^Glycobiology Unit, Institute of Reproductive Biology, FBN, Dummerstorf, Germany; ^6^Service Group Cytometry, Institute of Reproductive Biology, FBN, Dummerstorf, Germany; ^7^Molecular Biology and Fish Genetics, Faculty of Agriculture and Environmental Sciences, University of Rostock, Rostock, Germany

**Keywords:** gene expression profiling, handling stress, head kidney, maraena whitefish, microarray, phagocytosis

## Abstract

Aquaculture management involves regular handling procedures, but these can evoke stress responses in farmed fish. We compiled an extensive list of published parameters that indicate the most likely handling-induced physiological deviations from the norm. However, since these parameters are based almost exclusively on studies of rainbow trout and Atlantic salmon, we conducted a handling-challenge experiment with maraena whitefish (*Coregonus maraena*). This salmonid fish was sampled at either 3 or 24 h after a single 1-min handling or after 10 days of daily repeated 1-min handling. The cortisol levels were strongly elevated in some individuals at 3 h after the single handling challenge, but these elevations were not significantly different between the challenged and control cohorts. The phagocytic capacity of myeloid head-kidney cells stimulated with fluorophore-labeled, inactivated *Aeromonas salmonicida* was significantly decreased in maraena whitefish at 3 h after the handling challenge compared to control fish. Microarray analysis of head-kidney samples from the challenged and control fish revealed 12 differentially expressed genes at 3 h and 70 at 24 h after the single handling episode, but only 5 differentially expressed genes after 10 days of repeated daily handling. The identified genes were assigned to numerous stress- and immune-relevant functional pathways, including “glucocorticoid receptor signaling” (3 h post-challenge), “HIF1A signaling” (24 h post-challenge), or “complement system” (10 days of repeated challenge). Our data reveal the tight interconnection of immune and stress pathways in the head kidney of maraena whitefish and corroborate several parameters previously found regulated in other tissues of handling-stressed rainbow trout. These findings indicate that handling may compromise the health and welfare of maraena whitefish in aquaculture.

## Introduction

Maladaptive stress responses are associated with reprogramming of the immune system. A comprehensive panel of biomarkers provides a reliable source of information for evaluation of the actual immune status and the impact and severity of a particular environmental challenge. Biomarkers are substances or characteristics that indicate an upcoming or persistent condition (1), as they reflect the “interaction between a biological system and an environmental agent of chemical, physical, or biological origin” (2, 3). In non-mammalian model species, such as teleost fishes, gene profiling has been widely used to search for parameters that are sensitive to environmental disturbances and that might be suitable for use as potential diagnostic markers for disease and distress (4–6). Different methodologies allow the screening of either preselected genes (quantitative PCR, qPCR) or whole transcriptomes (microarray, RNA sequencing) in different tissues and/or under different challenge conditions. The simultaneous assessment of multiple parameters, preferably from different analysis platforms, allows the drawing of realistic conclusions about unsuitable husbandry conditions and environmental stressors that may exceed the adaptive capacity of the examined individuals (7, 8). The recording of gene expression markers in conjunction with other molecular markers, such as plasma hormones or metabolites, behavioral features, growth rates, feed intakes, or parasite infestations (7), can reflect potential alterations at the different levels of the fish's biological response (9, 10).

Stressors in aquaculture can impair the immune defenses of fish, thereby facilitating the appearance of diseases (11). Handling stress, in particular, has been intensively investigated in the salmonids rainbow trout *Oncorhynchus mykiss* and Atlantic salmon *Salmo salar*, as these species have been domesticated for decades and are therefore more resilient to anthropogenic stress (12–14). These studies have provided an extensive list of a wide variety of parameters that change after typical handling procedures, such as chasing and netting, exposure to air, and transfer to (confinement) tanks (Table 1). The investigated parameters include body scores (15), concentrations of osmolytes and metabolites (e.g., hormones, carbohydrates, lactate, nucleotides, free fatty acids, and sialic acids) (16–21), blood cell counts (16, 22), enzyme activities (23–25), behavioral characteristics (23), susceptibility to parasites (26), and a long and diverse list of tissue-specific transcripts recorded *via* qPCR (27–29) or transcriptomic approaches (30–34).

**Table 1:** Overview about selected physiological parameters altered in response to handling stress in *Oncorhynchus mykiss* and *Salmo salar*.

**Experimental handling procedure**	**Duration**	**Time of sampling after challenge**	**Selected indicative parameters**	**Direction**	**References**
***S. salar*** **:**					
Net confinement	9 min	1, 3, 5, and 8 h	*Metabolite concentration*: cortisol, glucose, free fatty acids, osmoles, sodium, chloride (plasma)	Up	(17)
Netting and transfer to low-water tanks	10 min	—	*Metabolite concentration*: inosine monophosphate (white muscle)	Up	(20)
Netting and transfer to low-water tanks	10 min	—	*Metabolite concentration*: adenosine-5'-triphosphate, phosphocreatine (white muscle)	Down	(20)
Daily chasing, netting, transfer to low-water tanks, and draining the tank	15 min each	42 days	*Metabolite concentration*: growth hormone, insulin-like growth factor (plasma)	Up	(15)
Twice daily chasing, netting, transfer to low-water tanks and draining the tank	15 min each	11 and 20 days	*Body score*: length, weight, condition factor	Down	(15)
Netting, exposure to air and transfer to low-water tanks	10 s air exposure	30 min	*Metabolite concentration*: cortisol (plasma), hemoglobin (blood); *Cell number*: heterophilic granulocytes, thrombocytes (blood)	Up	(22)
Netting, exposure to air and transfer to low-water tanks	10 s air exposure	30 min	*Cell number*: white blood cells, particularly lymphocytes (blood)	Down	(22)
Handling and exposure to air	15 s	1 h	*Metabolite concentration*: cortisol, glucose (plasma); *Transcript level*: interleukin-1-beta (head-kidney macrophages)	Up	(28)
Daily netting out of water	15 s	4 weeks	*Metabolite concentration*: di-O-acetylated sialic acids (plasma)	Up	(18)
Daily netting out of water	15 s	2 weeks	*Metabolite concentration*: carbohydrates, O-phosphocholine, lactate, alanine, valine, trimethylamine-N-oxide (plasma)	Up	(19)
Netting out of water	30 s	3 h	*Transcript level*: mucins (gill)	Up	(27)
Netting out of water	15 s	1 h	*Metabolite concentration*: cortisol (plasma); *Susceptibility*: attachment of copepodids	Up	(26)
***O. mykiss*** **:**					
“Hook and line” stress	2 min	20 min	*Metabolite concentration*: glucose (plasma); *Cell numbers*: thrombocytes, hematocrit (blood)	Up	(16)
“Hook and line” stress	2 min	10–60 min	*Enzyme activity*: clotting time	Down	(16)
Confinement in a shaking bucket	30 min	3 h	*Enzyme activity*: lysozyme (plasma, kidney); *Behavior*: opercular ventilation frequency	Up	(23)
Chasing	5 min	1 h	*Metabolite concentration*: cortisol, glucose (plasma)	Up	(21)
Chasing	5 min	2, 4, and 8 h	*Metabolite concentration*: growth hormone (plasma)	Down	(21)
Netting, transportation in a bucket, transfer to a new tank and chasing in the new tank	10 min	—	*Metabolite concentration*: cortisol, glucose (serum); *Enzyme activity*: lysozyme (serum)	Up	(24)
Daily netting	2 min	5 days	*Metabolite concentration*: cortisol (plasma); *Transcript levels*: cytochromes, glyceraldehyde-3-phosphate dehydrogenase, fructose-bisphosphatase aldolase A, glutamate carboxypeptidase, growth factor receptor-bound protein 2 -related adapter protein, transposase etc. (kidney)	Up	(30)
Daily netting	2 min	5 days	*Transcript level*: creatine kinase, microtubule-associated protein RP/EB, myosin chains, parvalbumin, sarcoplasmic reticulum calcium ATPase, troponin (brain)	Down	(30)
Netting, exposure to air and transfer to low-water tanks	30 s	3 h	*Metabolite concentration:* cortisol, glucose (plasma); *Transcript level*: C1q-like protein, complement receptor, glucose-6-phosphatase, MHC class 1 heavy chains, nuclear protein 1, transcription factor JunB, tumor necrosis decoy factor receptor (liver)	Up	(31)
Netting and chasing	3 min	1 h	*Metabolite concentration*: cortisol (plasma); *Transcript levels*: androgen receptor, arginase, ATPase B, cystatin, glucokinase, ubiquitin, cathepsin D, vitelline envelope proteins, glutamine synthetase 3, glucose transporter II, lipoprotein lipase, matrix metalloproteinase-1, nitric oxide synthase pyruvate kinase, cytochrome P450, retinal binding protein, SRY-related HMG-box transcription factors etc. (liver)	Up	(32)
Netting and chasing	3 min	24 h	*Transcript level*: aryl hydrocarbon receptor, solute carrier family 2, member 1, glutamate-ammonia ligase, 20-B-hydroxysteroid dehydrogenase, cytochrome monooxygenase 2k5, cathepsin D, insulin-like growth factor-2 etc. (liver)	Up	(32)
Transfer to confinement tanks	24 h	—	*Metabolite concentration*: adrenocorticotropic hormone, cortisol, glucose (plasma); *Transcript level*: calcium-transporting ATPase 2, complement factor H, damage-specific DNA-binding protein, fibrinogen chains, glucogen synthase kinase-binding protein, glutamine synthetase, haemoglobin beta-chain, haptoglobin, immunoglobulin epsilon Fc receptor, orosomucoid, serum albumin, tryptophanyl-tRNA synthetase (liver)	Up	(33)
Netting and a manual stripping procedure every 2 days	15 min	21 days	*Transcript level*: tumor necrosis factor (liver)	Up	(29)
Transfer to confinement tanks	3 h	—	*Transcript level*: barrier-to-autointegration factor, mitochondrial phosphate carrier member 25, diablo IAP-binding mitochondrial protein, chitinase 1, calcium-binding mitochondrial carrier protein, pepsinogen c, coenzyme q-binding protein coq10, regulator of G-protein signaling 1, insulin-like growth factor binding proteins, papilin, transcription factor cp2, transmembrane protein 80, tsc22 domain member 3, zymogen granule membrane protein 16 etc. (liver)	Up	(34)
Transfer to confinement tanks	3 h	—	*Transcript level*: arrestin domain containing protein 3, c1q-like protein 4, C-type lectin domain family 4, member E enolase 2, G-protein-coupled signaling factor, heat-shock proteins, kit receptor, reproduction regulator 2, MHC class II alpha chain, multidrug and toxin extrusion protein 1, neurofilament polypeptides, proto-oncogene c-fos, tubulin, etc. (liver)	Down	(34)
Repeated chasing	5 min	45 min	*Metabolite concentration*: lactate, triglycerides (plasma); *Enzyme activity*: 3-hydroxyacyl-coenzyme A dehydrogenase, glucocorticoid receptor 1 and 2, glucose 6-phosphatase (liver); *Transcript levels*: 6-phosphatase and glucose transporter 2 (liver)	Up	(25)
Repeated chasing	5 min	45 min	*Metabolite concentration*: fatty acids (plasma), glycogen (liver); *Enzyme activity*: fatty acid synthase (liver); *Transcript levels*: glucokinase, pyruvate kinase, phosphoenolpyruvate carboxykinase (liver)	Down	(25)

**Figure 1:**
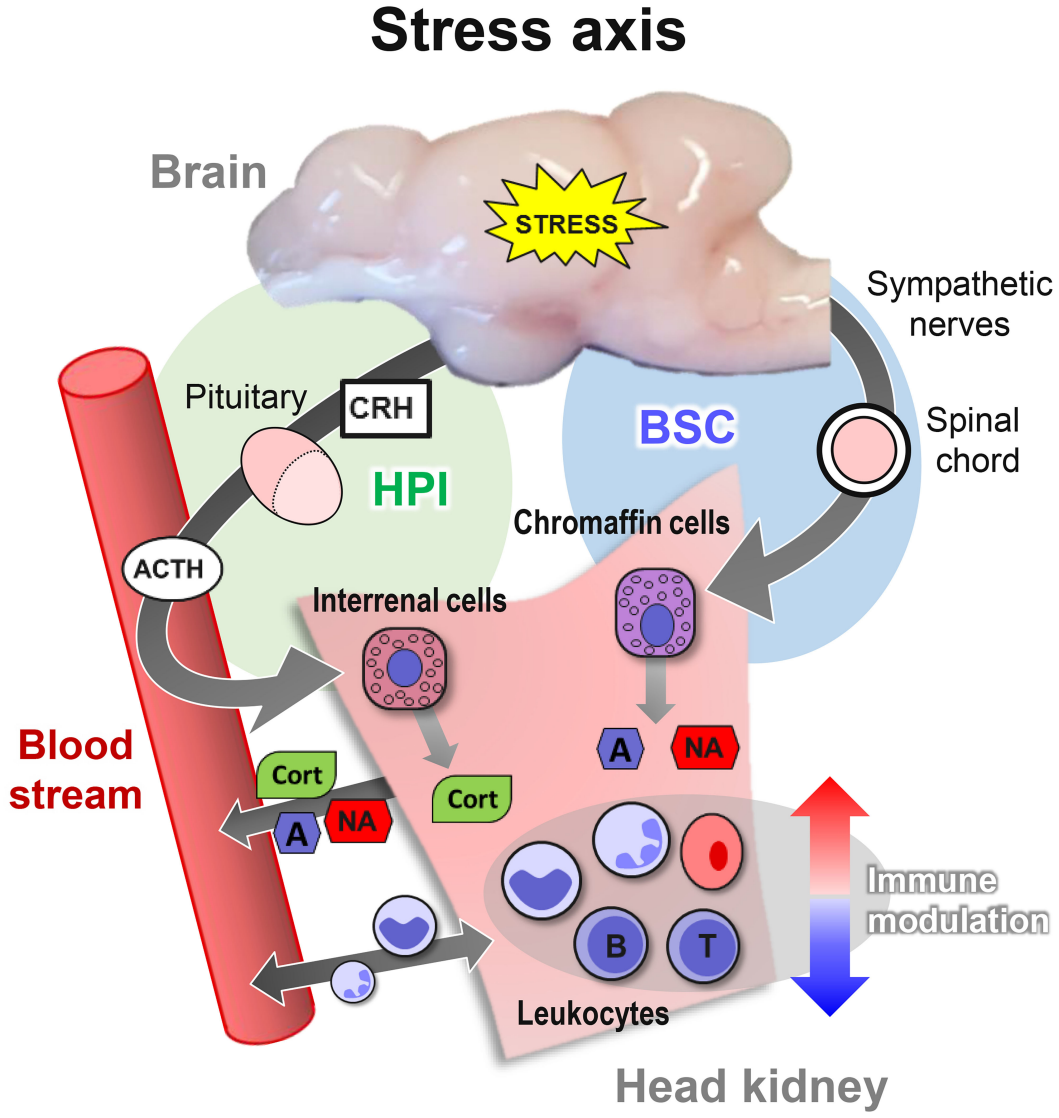
Overview of the three levels of the stress response in teleost fish. The primary response activates the brain-sympathetic-chromaffin (BSC) axis. Sympathetic nerve fibers transfer the stress signals through the spinal cord and the sympathetic ganglia, which innervate and form synapses with chromaffin cells in the head kidney. The synapse activity triggers the release of the catecholamines adrenaline (A) and noradrenaline (NA) into the bloodstream. In parallel, the activation of the hypothalamic–pituitary–interrenal (HPI) axis stimulates the release of corticotropin-releasing hormone (CRH) and adrenocorticotropic hormone (ACTH) into the blood. ACTH, in turn, induces the interrenal cells in the head kidney to release cortisol (CORT) into the bloodstream. CORT promotes the secondary response of reorganizing the availability of energy resources, as reflected by altered blood pressure and heart rate and by the changed concentrations of glucose and lactate. The tertiary response is reached when the system assumes a dysregulated state but cannot return to homeostasis, thereby impairing the immune system, behavior, growth, and reproduction.

By contrast, other salmonid species, such as the maraena whitefish *Coregonus maraena*, are non-domesticated, as they have been kept in aquaculture for only a few generations and on a comparatively small scale (35). These other fish species respond inappropriately to unavoidable aquaculture procedures, including transportation and tank cleaning, and only sparse reports are available regarding their physiological reaction patterns. However, a common response among all vertebrates is the immediate activation of the stress axis by an adequate stimulus. This activation is initiated in the brain and leads to the release of stress hormones into the bloodstream by specialized endocrine tissues (Figure 1). The chromaffin and interrenal cells of the head kidney, in particular, are in charge of secreting catecholamines and glucocorticoid hormones (e.g., cortisol), the main endocrine effectors of the stress response (11, 36, 37).

In a previous study, we analyzed the monoaminergic response to acute and repeated exposure to handling in the brain of maraena whitefish (38). In the present study, we recorded the effect of the same challenge, but with a focus on the phagocytic capacity of myeloid head-kidney cells and the transcriptomic response of the head kidney.

## Materials and Methods

### Animal Rearing Conditions

Juvenile maraena whitefish were obtained from the Institute for Fisheries of the State Research Center for Agriculture and Fishery Mecklenburg-Western Pomerania (Born, Germany) and BiMES—Binnenfischerei GmbH (Friedrichsruhe, Germany). Following transportation, the maraena whitefish were habituated for 2 weeks in fresh-water recirculation systems at 18°C at a stocking density of 10 kg/m3 and a 12:12-h day-night cycle. The quality of the water was sustained by automated purification and disinfection (a bio-filter and UV light), with continuous recording of relevant quality parameters ($\text{N}{\text{H}}_{4}^{+}$, $\text{N}{\text{O}}_{2}^{-}$, $\text{N}{\text{O}}_{3}^{-}$, NH_3_; pH value; temperature and oxygen saturation). Commercial dry feed pellets (4.5 mm; Biomar, Inicio Plus) were distributed by automatic feeders at a daily rate of 0.8–4.0%, depending on the weight of the animals.

### Handling Experiment With Maraena Whitefish

**Figure 2:**
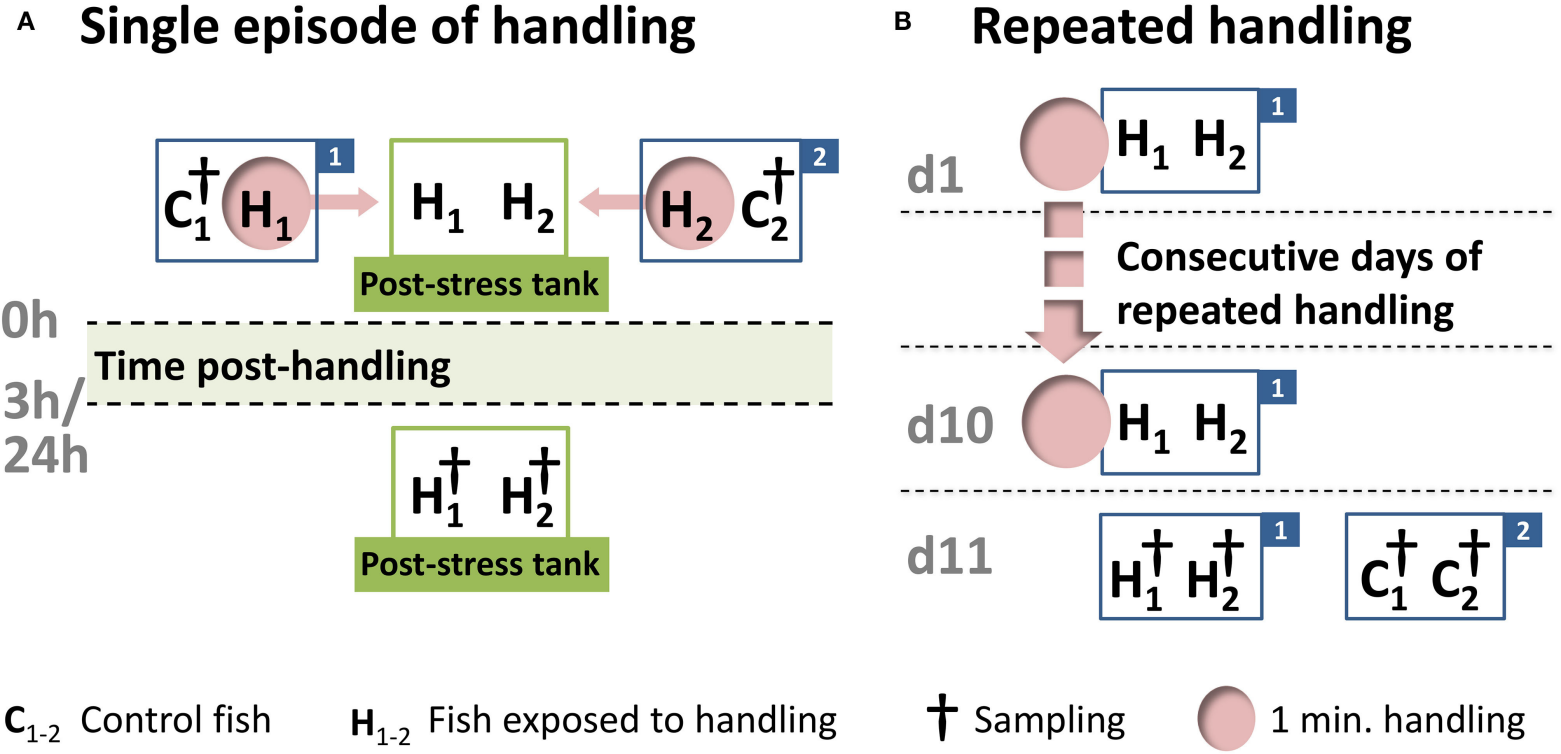
Schematic representation of the handling experiment. After a 7-day acclimation period in the experimental tanks, 24 maraena whitefish were exposed to a one-minute handling challenge (indicated by “H” and pink circles), while 24 control fish remained undisturbed (indicated by “C”). **(A)** The single handling challenge involved chasing, netting, and the transfer from one tank (labeled with “1” or “2”) to another tank (termed the “post-stress tank”). Sampling (indicated by †) was conducted at 3 or 24 h after the handling challenge. This handling-challenge experiment was repeated four times, starting on different days, with rotation of the experimental tanks to avoid tank effects. A total of 16 fish were sampled per time point. **(B)** The repeated handling challenge was performed every morning in the same tank (labeled “1”), while the control fish (in tank “2”) remained undisturbed. After 10 days, the treated and control fish were sampled (indicated by †). This experiment was conducted four times (16 fish in total), starting on different days and with rotation of the experimental tanks to avoid tank effects.

One week before the start of the experiments, 48 juvenile whitefish, with a start length of about 20 cm and a start weight of about 200 g, were acclimatized to dark cylindrical 150 L polyethylene tanks. The present study involved a single handling challenge for the maraena whitefish, followed by sampling after 3 h (*n* = 8) and 24 h (*n* = 8) or repeated daily handling over a period of 10 days (*n* = 8) (Figure 2). The fish were kept in pairs in separate tanks, as described in detail previously (38). For the single 1-min handling challenge, the 16 fish were intermittently chased and netted for 1 min. They were then lifted separately from the tank and transferred into a secondary tank, where they were kept until the 3 or 24 h post-handling time point. The fish sampled at 3 and 24 h were kept in different tanks. The repeated 1-min handling procedure was applied on a daily basis for a 10 day period, and the 8 treated fish were sampled at 24 h after the last handling treatment. Cohorts of control fish (24 fish in total) were kept in parallel under identical conditions but not exposed to the handling challenge.

Experiments were performed in the morning (between 8 and 11 a.m.) to minimize the influence of circadian rhythms on the measurements. All procedures were approved by the Landesamt für Landwirtschaft, Lebensmittelsicherheit und Fischerei, Mecklenburg-Vorpommern, Germany (LALLF M-V/TSD/7221.3-1-069/18). The sampling and killing methods followed the standards described in the German Animal Welfare Act [§ 4(3) TierSchG]. In detail, whitefish were euthanized with an overdose of 2-phenoxyethanol (0.7 mL/1 L water), followed by spine sectioning at the skull level. Immediately after euthanasia, blood was extracted from the caudal vein using a heparinized syringe and stored in heparinized vials on ice. The head kidneys were then isolated, as the head kidney is the effective center for neuro-immune interactions in teleosts and controls endocrine, hematopoietic, and immune functions (39, 40). Half of the tissue was snap-frozen in liquid nitrogen (for microarray hybridization), and the other half was placed in a sterile container with Dulbecco's Modified Eagle's Medium (DMEM; Gibco/Thermo Fisher) for cell extraction for the phagocytosis assay and cell sorting.

### Measurement of Blood Parameters

Glucose and lactate concentrations in freshly extracted blood were analyzed using a portable reflectance photometry device (Accutrend Plus, Roche) and the respective test strips (Accutrend BM, Roche).

Plasma was extracted from heparinized blood by centrifugation at 6°C and 270 × g for 15 min. The erythrocyte pellet was discarded, and the plasma was used to quantify the cortisol concentrations using a competitive enzyme-linked immunosorbent assay (ELISA; DRG Diagnostics). A Beckman Coulter DTX 800/880 Series Multimode Detector (Beckman Coulter) detected the level of absorbance at 450 nm. Measurements were performed following the manufacturer's instructions.

### Isolation of Head-Kidney Cells and Phagocytosis Assay

The effects of handling on the phagocytic activity of myeloid cells were quantified using samples from fish treated with “single 1-min handling” and sampled 3 h post-treatment (*n* = 4) and in samples from matching controls (*n* = 4). The isolation of head-kidney cells followed our previously published protocols (40). In brief, head-kidney tissue was homogenized in DMEM using a steel sieve (500 μm). The resulting head-kidney cell suspension was then successively sieved through two cell strainers (200 and 100 μm pore size). The sieved cell suspension was then layered onto an isotonic Percoll gradient (ρ = 1.084 g/mL) and centrifuged at 800 × g for 30 min to reduce the number of erythrocytes. The cell band in the DMEM/Percoll interface was collected, washed, and resuspended in DMEM to obtain a final concentration of 20 million cells per mL. A 100 μL sample of this cell suspension was incubated with or without fluorescein isothiocyanate (FITC; Miltenyi Biotec) labeled *Aeromonas salmonicida* (wild-type strain JF 2267) at a 2.5:1 (head-kidney cell:bacteria) ratio at 17°C for 30 min. The cell suspension was then analyzed by fluorescence activated cell sorting using a MoFlo XDP high-speed cell sorter (Beckman Coulter) with an incorporated air-cooled Coherent Sapphire laser (488 nm, 100 mW) to identify the cells of myeloid origin [larger cells with high internal complexity; *cf*. (40)]. This cluster of cells was sorted and reanalyzed with the cell sorter to quantify the FITC-positive and FITC-negative cells inside the cluster. A Carl Zeiss confocal laser-scanning microscope LSM 800 (Carl Zeiss Microscopy) with a Plan-Neofluar 40 × /1.30 oil objective (Carl Zeiss Microscopy) was used to validate the internalization of FITC-labeled bacteria by myeloid cells and to discard aggregates of fluorescent bacteria, which could be recorded as false positives.

The same animals used for the phagocytosis assay were selected for microarray analysis.

### RNA Isolation and Microarray Analysis

Total RNA was extracted from head-kidney samples by homogenization in liquid nitrogen using TRIzol Reagent (Life Technologies/Thermo Fisher) and the RNeasy Mini Kit (Qiagen). The concentration of the obtained RNA was determined by spectrophotometry (NanoDrop One, Thermo Fisher Scientific) and its quality was assessed by horizontal electrophoresis on 1% agarose gels. The integrity of the RNA was analyzed using the Bioanalyzer (Agilent), which revealed RNA integrity numbers > 9 for all samples analyzed.

Hybridizations were carried out using 8 × 60 k Agilent-020938 Salmon Oligo Microarrays (Agilent Technologies; GEO platform: GPL21057) that included the transcript-fragment sequences from *Oncorhynchus, Salmo*, and *Coregonus* spp. (41). Samples (50 ng) of the extracted RNA specimens (four controls and four treated fish for each 3 h, 24 h, and 10 day time point) were converted first to Cy3-labeled cRNA using a Low Input Quick Amp Labeling Kit (Agilent Technologies). The ND-1000 Spectrophotometer was used to measure the cRNA yield and dye-incorporation rate. The hybridization was performed using the Agilent Gene Expression Hybridization Kit (Agilent Technologies) according to the One-Color Microarray-Based Gene Expression Analysis protocol (version 6.6, part number G4140-90040). In detail, the Cy3-labeled fragmented cRNA in a hybridization buffer was hybridized overnight (17 h, 65°C) to two 8 × 60 k Agilent-020938 Salmon Oligo Microarrays using Agilent's hybridization chamber and oven. When hybridization was complete, the microarray chips were washed with Agilent Gene Expression Wash Buffer 1 (room temperature, 1 min), followed by a second wash with the preheated Agilent Gene Expression Wash Buffer 2 (37°C, 1 min). The signaling fluorescence of the hybridized arrays was scanned using Agilent's Microarray Scanner System G2505C (Agilent Technologies) at a resolution of 2 μm.

### Data Analysis

Statistical analyses of blood parameters were performed using GraphPad Prism 9.1.0 (GraphPad Software) with a significance level of 0.05.

The Agilent Feature Extraction software (version 12.1.1.1) was used to read and process the microarray image files. Data were normalized by subtracting the background fluorescence based on a two-sided *t*-test analysis. The resulting data sets were analyzed in RStudio (v. 1.4.1106) for R (v. 3.6.3) with the limma package from the Bioconductor suite. The expression fold-change (FC) between treated fish and controls was calculated, and Benjamini and Hochberg correction was applied to the *p*-values to minimize the false discovery rate. Genes were considered significantly expressed when *q* < 0.01 and absolute FC ≥ 1.75.

The Basic Local Alignment Search Tool (BLAST) was used to annotate the significantly expressed genes based on a coverage and sequence identity of >80% and *E*-value <1 × 10^−4^. The expression data have been submitted to Gene Expression Omnibus (GEO) under accession code GSE183125. The heat map was developed using RStudio, applying the functions gplot, tidyverse, cluster, factoextra, and pheatmap (https://www.bioconductor.org/). The list of differentially expressed (DE) genes (excluding repeated annotations) was used for a functional analysis with the Ingenuity program (Ingenuity Pathway Analyses, Ingenuity Systems/Qiagen). Benjamini-Hochberg multiple testing was performed, and *p* < 0.05 was considered as a cut-off score. The output lists were carefully reviewed, and all pathways and functions associated with a mammalian disease were deleted. All functionally relevant pathways are indicated by italics in the following sections.

## Results

### Cortisol Is Not Significantly Elevated in Whitefish 3 h After Handling

**Figure 3:**
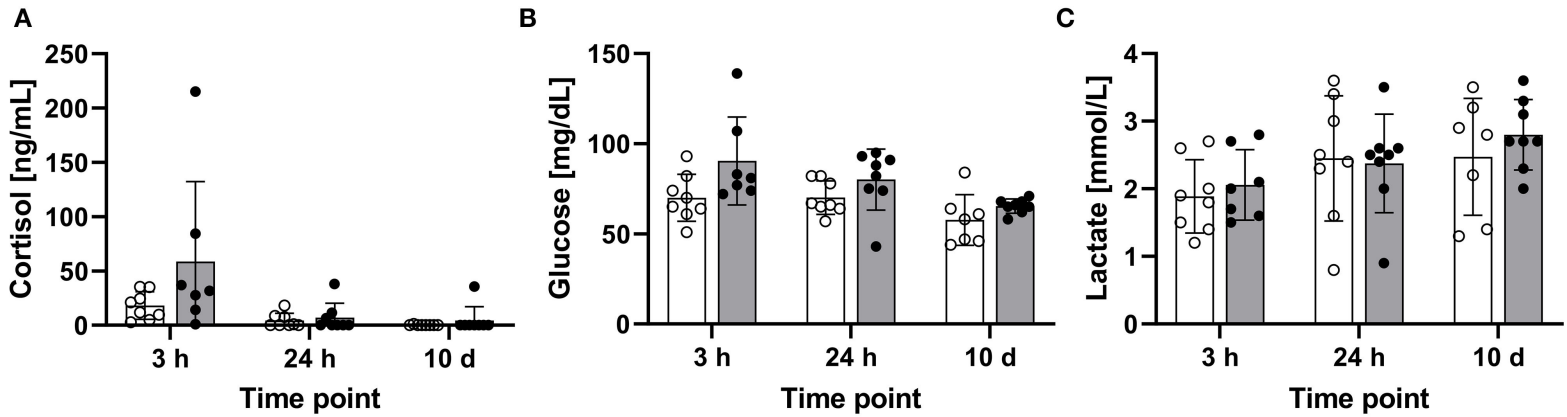
Concentrations of blood parameters in the treated and control maraena whitefish. The concentrations of **(A)** plasma cortisol (ng/mL), **(B)** glucose (mg/dL), and **(C)** lactate (mmol/L) in eight individuals per time point are illustrated as box and whisker plots. Bars represent the mean values in control (white) and treated (gray) fish. Individual measurement points are indicated by blank (control) and filled (treatment) dots; error bars indicate standard deviation.

The blood concentrations of cortisol, glucose, and lactate were determined in the treated maraena whitefish and matching controls 3 and 24 h after a single episode of 1-min handling and after a repeated 1-min handling challenge over 10 days. The cortisol concentrations in some individuals reached values of up to 215 ng/mL at 3 h after the handling challenge, but the cortisol level elevations were not consistent across the entire cohort. Therefore, no significant differences were detected at other time points or with the matching controls (Figure 3A). The levels of glucose (43–139 mg/dL) and lactate (0.8–3.6 ng/mL) also did not differ significantly between the investigated groups (Figures 3B,C).

### Single and Repeated Episodes of Handling Result in Moderate Changes in the Head-Kidney Transcriptome

**Figure 4:**
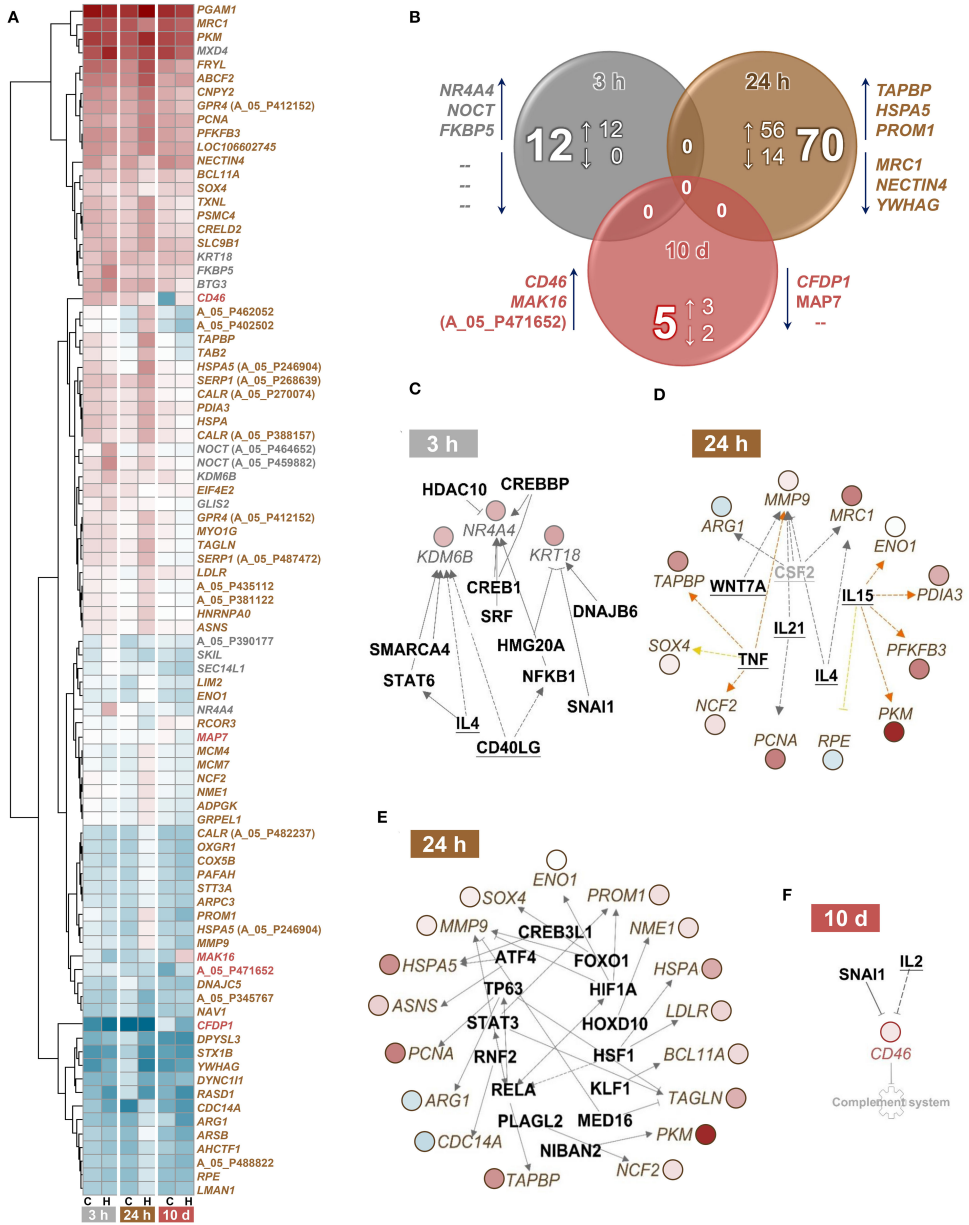
Classification of differentially expressed (DE) genes in treated vs. control maraena whitefish. **(A)** Hierarchical representation of DE features (*q* < 0.01; FC ≥ |1.75|) in the head kidney of maraena whitefish at 3 and 24 h after single handling and 10 days after repeated daily handling (as indicated below the heat map). The intensity and shade of the colored cells represent the normalized mean values of four individuals per group (C, control; H, handling) and time point (3 and 24 h after single handling; 10 days after repeated daily handling). Gene symbols or Agilent IDs (in cases of unknown features) are listed on the right and labeled according to the sampling time point at which differential expression over control was detected (3 h, gray; 24 h, brown; 10 d, red). **(B)** Venn diagram illustrating the absolute number and the number of upregulated (↑) and downregulated (↓) DE features in the head kidney of maraena whitefish 3 and 24 h after single handling and 10 days after repeated daily handling relative to the expression values obtained for the matching control group. The overlaps indicate that no DE genes are shared by the three treatment groups. The diagram was calculated on the basis of the Agilent ID lists; the three most upregulated and downregulated genes in the respective lists are given next to the respective circle. **(C–F)** Upstream analyses predicted cytokines (bold, underlined) and transcription factors (bold) that might be responsible for the renal expression patterns **(C)** at 3 h and **(D,E)** 24 h after single handling and **(F)** at 10 days after repeated daily handling of maraena whitefish. The gray factor is not present as a teleost ortholog. The observed upregulation and downregulation of the italicized DE genes is highlighted according to the colors of the heatmap in **(A)**. Relationships between upstream regulators and genes are displayed by lines indicating activation (orange) or uncertainty due to lack of knowledge (gray) or the state of downstream factors (yellow). Broken and solid lines indicate indirect or direct interactions; a blocked line indicates inhibition.

A single episode of handling had moderate effects on the head-kidney transcriptome of maraena whitefish (Figures 4A,B). The transcript abundances of a dozen genes were increased at least 1.75-fold (with *q* < 0.01) in whitefish 3 h after the challenge compared to the control fish (Figure 4A; GEO accession ID: GSE183125). A pathway analysis application allocated two of the 12 genes (*FKBP5* and *KRT18*) to *glucocorticoid receptor signaling* (with *p* = 0.01) (Table 2). An upstream analysis suggested two cytokines and ten transcription factors that could be responsible for the induction of three differentially expressed (DE) genes (*NR4A4, KDM6B*, and *KRT18*) (Figure 4C).

**Table 2:** Relevant Ingenuity-predicted canonical pathways (with *p* ≤ 0.01) regulated in challenged maraena whitefish.

**Canonical pathway**	**Number of included genes**	***p*-value**	**Involved DE genes**
**3 h:**
Glucocorticoid receptor signaling	333	0.01	*FKBP5, KRT18*
**24 h:**
HIF1A signaling	210	0.00002	*HSPA5, HSPA, MMP9, NCF2, PKM, RASD1*
Glycolysis	24	0.00004	*ENO1, PGAM1, PKM*
Antigen presentation pathway	38	0.00017	*CALR, PDIA3, TAPBP*
Synaptogenesis signaling pathway	307	0.00024	*ARPC3, DNAJC5, HSPA, PAFAH1B1, RASD1, STX1B*
Unfolded protein response	55	0.00052	*CALR, HSPA5, HSPA*
Cell cycle control of chromosomal replication	56	0.00055	*MCM4, MCM7, PCNA*
Steroid hormone signaling in epithelial cells	154	0.00098	*DNAJC5, HSPA5, HSPA, PDIA3*
Endoplasmic reticulum stress pathway	21	0.0016	*CALR, HSPA5*
Asparagine biosynthesis	1	0.0029	*ASNS*
Role of protein kinase receptor in interferon induction and antiviral response	114	0.0043	*HSPA5, HSPA, TAB2*
Signaling in epithelial cells and neutrophils	111-115	<0.0044	*ARPC3, NCF2, RASD1*
14-3-3-mediated/p70S6K signaling	126-128	<0.0059	*PDIA3,RASD1,YWHAG*
Protein ubiquitination pathway	268	0.0072	*DNAJC5, HSPA5, HSPA, PSMC4*
Phagosome maturation	141	0.0077	*CALR, DYNC1I1, NCF2*
Glutathione redox reactions	4	0.01	*PDIA3*
Arginine degradation	4	0.01	*ARG1*
**10 d:**
Complement system	36	0.0071	*CD46*

Sampling at 24 h after the handling challenge increased the number of DE genes to 70 (56 upregulated and 14 downregulated genes). These DE genes were assigned to 16 relevant pathways (with *p* ≤ 0.01), including the stress-related pathways *HIF1A signaling, unfolded protein response, endoplasmic reticulum stress pathway*, and *protein ubiquitination pathway*; the metabolic pathways *glycolysis, asparagine biosynthesis*, and *glutathione redox reactions*; and the immune-relevant pathways *antigen presentation pathway, role of protein kinase receptor in interferon induction and antiviral response*, and *arginine degradation* (Table 2). The upstream analysis suggested six cytokines (Figure 4D) and 14 transcription factors that may be responsible for the induction of 21 DE genes (Figure 4E).

The repeated handling over 10 days modulated the transcript levels of only five (three upregulated and two downregulated) genes, including *CD46* in the head kidney of maraena whitefish. *CD46* was the most upregulated gene (6.5-fold) of the entire study across all time points (Figure 4A). A pathway analysis suggested the cytokine IL2 and the transcription factor SNAI1 as negative upstream regulators of *CD46* expression (Figure 4F), which is predicted to control the *complement system* (with *p* = 0.007) (Table 2).

### Phagocytic Activity of Myeloid Cells Is Reduced 3 h After Handling

**Figure 5:**
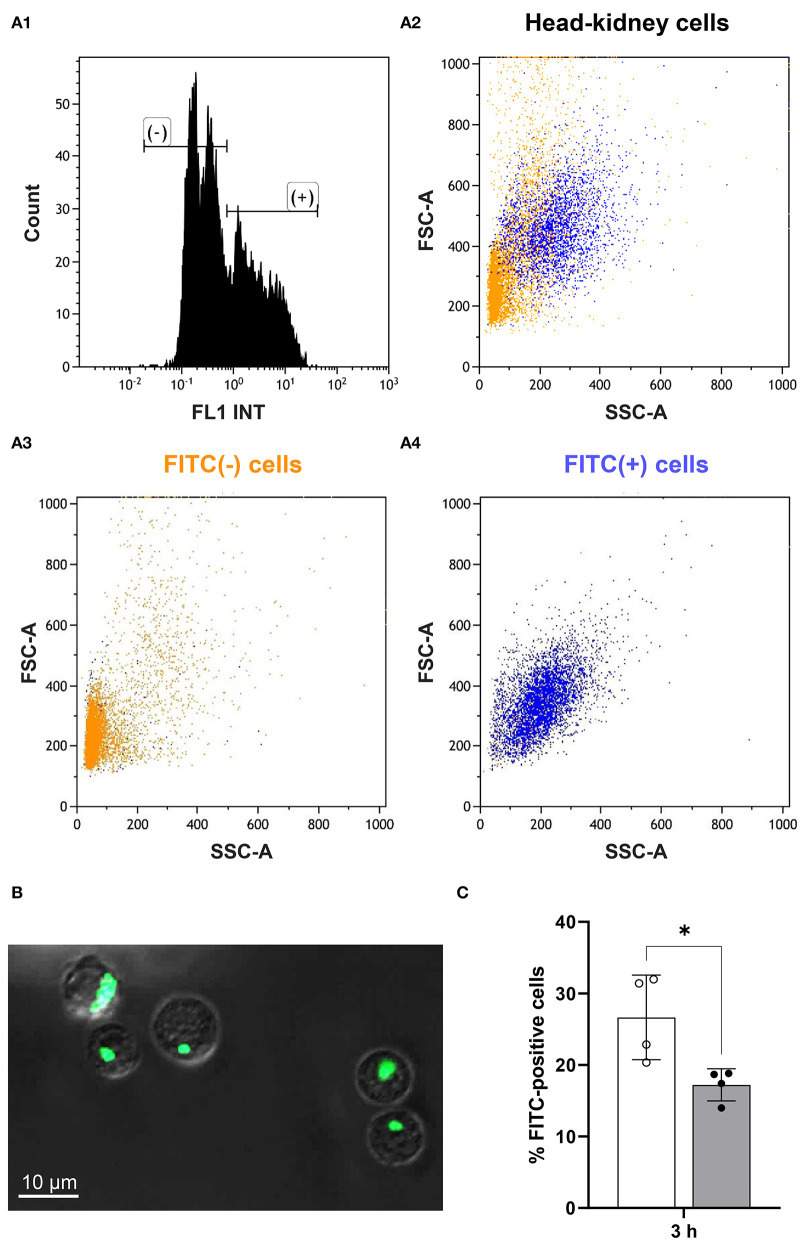
Phagocytic capacity of myeloid cells from treated and control maraena whitefish. Sorted myeloid cells were incubated with FITC-labeled *Aeromonas salmonicida*. A high-speed cell sorter separated and quantified the FITC-positive and FITC-negative cells. **(A1)** The histogram shows the quantitative distribution of FITC-positive (+) and FITC-negative (–) cells. **(A2–4)** The FSC-A/SSC dot plots illustrate the proportion of FITC (+) (blue cloud) and FITC (–) (orange cloud) cells. These cells were sorted according to the parameters of cell volume (abscissa) and granularity (ordinate); therefore, the orientation of the dot cloud in the diagram suggests that most of the phagocytosing cells are more granular and larger cells of myeloid origin [see (40) for further information]. **(B)** Confocal microscopy was used to validate the phagocytosis of labeled bacteria by myeloid cells. A representative picture shows FITC-labeled *A. salmonicida* (bright green) phagocytosed by myeloid cells (characterized by their high internal complexity). Scale bar represents 10 μm. **(C)** The percentages of FITC-positive cells from maraena whitefish 3 h after a handling challenge (gray bar, *n* = 4) and from control fish (white bar; *n* = 4) are represented by box and whisker plots; error bars indicate standard deviations. * indicates a significantly different cell number (with *p* = 0.02).

At 3 h after handling, we incubated head-kidney cells from challenged and matching control fish with FITC-labeled *Aeromonas salmonicida* and determined the ratio of FITC-positive and FITC-negative cells (Figure 5A) in the cluster of presumably myeloid cells. Confocal microscopy was used to validate the phagocytosis of labeled bacteria by the myeloid cells (Figure 5B), as this microscopy mode allows the identification of aggregates of fluorescent bacteria that may otherwise distort the results. The phagocytic capacity of myeloid cells from maraena whitefish was significantly decreased by 37% (with *p* = 0.02) at 3 h after handling compared with the undisturbed control fish (Figure 5C).

## Discussion

The stress-induced reprogramming of the immune system may be reflected by delayed wound healing, accelerated pathogenesis, decreased vaccine protection, and modulated autoimmune diseases (42, 43). In the teleostean head kidney, the neuroendocrine “stress” axis and the immune system are interlinked by several hormones, including adrenaline, noradrenaline, and the glucocorticoid cortisol, which are released by the chromaffin and interrenal cell populations of this organ. In the present study, we observed elevated cortisol levels in only a few maraena whitefish individuals at 3 h after the handling challenge, and we interpret this as possibly a subsiding cortisol response. Other researchers have made similar observations in salmonid fish after handling stress (21, 25, 33), giving us reason to assume that the cortisol levels in our fish had likely peaked earlier than 3 h post-challenge. Nevertheless, the pathway analysis of differentially regulated genes in maraena whitefish 3 h after the challenge suggests an activation of *glucocorticoid receptor signaling*, which is broadly considered as anti-inflammatory, antiproliferative, and proapoptotic (44).

The release of stress hormones initiates a cascade of secondary messengers, which alters the expression of a panel of genes (45, 46). These expression patterns also modulate the function of immune cells, including monocytes/macrophages, granulocytes, and lymphocytes, in the head kidney (47–49). For this reason, we investigated the impact of the experimental handling procedure on the transcriptome of the head kidney and the consequences of handling on innate immune functions in maraena whitefish. Our list of DE genes at 24 h after handling shares a remarkable degree of overlap with the previously published lists on handling-induced expression profiles (30–34), despite the previous use of tissues other than the head kidney from the related species rainbow trout. Microarray studies on the liver (32) or brain (30) of handling-stressed rainbow trout revealed increased transcript levels of genes coding for matrix metalloproteinases (MMP), arginase (ARG), SRY-box transcription factors (SOX), the heat-shock gene *DNAJC5*, and four genes associated with myosin (MYO), which we also found upregulated 24 h after handling in the head kidney of maraena whitefish. The upregulation of nine features associated with MHC-class I molecules in the liver of handling-stressed rainbow trout (31) agrees with the increased levels of *TAPBP* (encoding the MHC-class I antigen-processing molecule tapasin) in the present study. The increased hepatic expression of genes coding for a G-protein-coupled signaling factor in an RNA-seq analysis of handling-stressed rainbow trout (34) corresponds to the upregulation of *GRP4* in maraena whitefish. These DE genes could prove to be suitable biomarkers for evaluating inappropriate handling of salmonids and for monitoring the advances in domestication processes of salmonid species.

The transcriptional response to the handling procedure increased six-fold after 24 h (compared to the 3 h sampling made 21 h earlier) and included numerous genes that contribute to both stress and immune pathways. The pathway *steroid hormone signaling in epithelial cells* likely reflects the glucocorticoid-mediated physiological changes and is closely interrelated with the canonical “stress pathways” *HIF1A signaling, unfolded protein response, endoplasmic reticulum stress pathway*, and *protein ubiquitination pathway*. The restructuring of metabolic processes is reflected by differentially regulated pathways, such as *glycolysis*, that mobilize energetic resources. The pathways *arginine degradation* and *signaling in epithelial cells and neutrophils* indicate the proliferation and/or differentiation of phagocytic immune cells, such as macrophages and neutrophilic granulocytes. In this context, the pathways *antigen presentation pathway, glutathione redox reactions*, and *phagosome maturation* suggest that handling affects key processes involved in the detection and destruction of potentially invading pathogens. In line with this prediction, we observed a significant decrease in phagocytosis by the phagocytic cells from treated whitefish compared with the undisturbed controls. The finding of decreased phagocytosis capacity is worrisome because it may indicate an increased susceptibility to bacterial infection. The resistance to pathogens is apparently weakened in teleost fish following a disruptive stimulus, as has been concluded by other authors (50–52). Similar conclusions were drawn from studies that investigated the relationship between handling stress and parasite infestation (26).

The interconnection of stress and immune pathways early after the onset of a disturbing stimulus is also illustrated by the cytokines and transcription factors that were predicted to have caused the altered expression profiles in the treated maraena whitefish. At all the time points examined, members of the interleukin-2 family (IL2, IL4/13, IL15, and IL21) were presumably involved in the regulation of the handling-induced expression profiles. The respective interleukins are considered to share conserved pleiotropic functions with their mammalian orthologs (53); this would include the differentiation of T cells and B cells, which develop in the head kidney. Stress has been reported to influence the ratio of leukocyte populations in fish blood (7, 54, 55) and the results of our pathway analysis suggest that leukocyte proliferation was modulated in response to handling.

Notably, tumor necrosis factor alpha (TNF) presumably contributed to the expression profile at 24 h after the single handling. TNF is an early cytokine that emerges after a disruptive stimulus in various fish species (56). The list of transcription factors with predicted involvement in the expression profiles at 3 h and 24 h after handling included both typical stress-related factors (e.g., HSF1, CREB1, and ATF4) and classical immune-related factors (mainly STATs and Rel/NF-κB). FOXO1 is a transcription factor that regulates the availability of energy and the type of antibodies produced by B cells, so it may also have an ambivalent role in stress and immune pathways.

After 10 days of repeated daily handling, only a few genes were differentially regulated relative to undisturbed control fish. One of these was *CD46*, which encodes a transmembrane glycoprotein that protects cells from excessive complement activation in many vertebrates, including fish (57). The activity and expression levels of complement components in salmonid fish are induced by husbandry practices and environmental stress (58–60), and transcriptomic analyses on handling-stressed rainbow trout have also identified complement genes (complement factor H, C1q-like proteins, complement receptor) as regulated features (31, 33, 34).

The low number of differentially regulated genes after 10 days of repeated handling agrees with our previously reported results (38). The lack of changes in plasma cortisol concentrations and monoamine activities in the brain over a 10 day period of repeated handling suggested a habituation to handling procedures over time. The genes encoding dopamine receptors and monoamine synthesis enzymes could therefore be potential indicators of habituation in the brain. The present study extends the panel of indicators to additional genes in the head kidney and confirms that maraena whitefish might habituate to the daily handling conditions applied in this study.

## Conclusion

A comprehensive panel of biomarkers provides a reliable source of information for evaluation of the actual biological status of fish and the impact and severity of a particular environmental challenge. This information is especially important for species that have only recently been introduced into intensive aquaculture and have not yet undergone breeding programs. A technical approach based on global transcriptome analysis and complemented by the quantification of metabolite-based parameters has not yet been published for coregonid species. The handling challenge tested in this study had already reduced the phagocytic activity of myeloid cells from maraena whitefish at only 3 h after the treatment, and this could increase disease susceptibility. The level of various genes involved in stress and immune pathways deviated from the norm 24 h after the challenging event. However, 10 days of repeated handling resulted in modulation of the concentrations of significantly fewer parameters. Taken together, the data indicate that relatively short handling procedures have a measurable impact on the physiology of maraena whitefish and should be minimized as much as possible to prevent increased disease susceptibility. Our data also show that habituation may decrease the activation of the stress axis and subsequent immune suppression. Therefore, close monitoring of the wellbeing of fish in aquaculture is essential to establish a balance between challenges that cause distress and subtler challenges that can lead to habituation.

## Data Availability Statement

The datasets presented in this study can be found in online repositories. The names of the repository/repositories and accession number(s) can be found below: https://www.ncbi.nlm.nih.gov/geo/, GSE183125.

## Ethics Statement

The animal study was reviewed and approved by Landesamt für Landwirtschaft, Lebensmittelsicherheit und Fischerei, Mecklenburg-Vorpommern, Germany. Written informed consent was obtained from the owners for the participation of their animals in this study.

## Author Contributions

AR, UG, JM-R, and TG designed research. JM-R, RB, and MT performed stress experiments and sampled fish. JM-R, TV, and UG performed phagocytosis assays and flow cytometry analyses. DK, AR, and JM-R conducted microarray analyses. JM-R and AR performed statistical analyses and wrote the paper. UG reviewed and edited the manuscript. All authors have read and agreed to the final version of the manuscript.

## Funding

JM-R has been funded by an inter-institutional PhD project of the FBN. The publication of this article was funded by the Open-Access Fund of the FBN.

## Conflict of Interest

The authors declare that the research was conducted in the absence of any commercial or financial relationships that could be construed as a potential conflict of interest.

## Publisher's Note

All claims expressed in this article are solely those of the authors and do not necessarily represent those of their affiliated organizations, or those of the publisher, the editors and the reviewers. Any product that may be evaluated in this article, or claim that may be made by its manufacturer, is not guaranteed or endorsed by the publisher.
